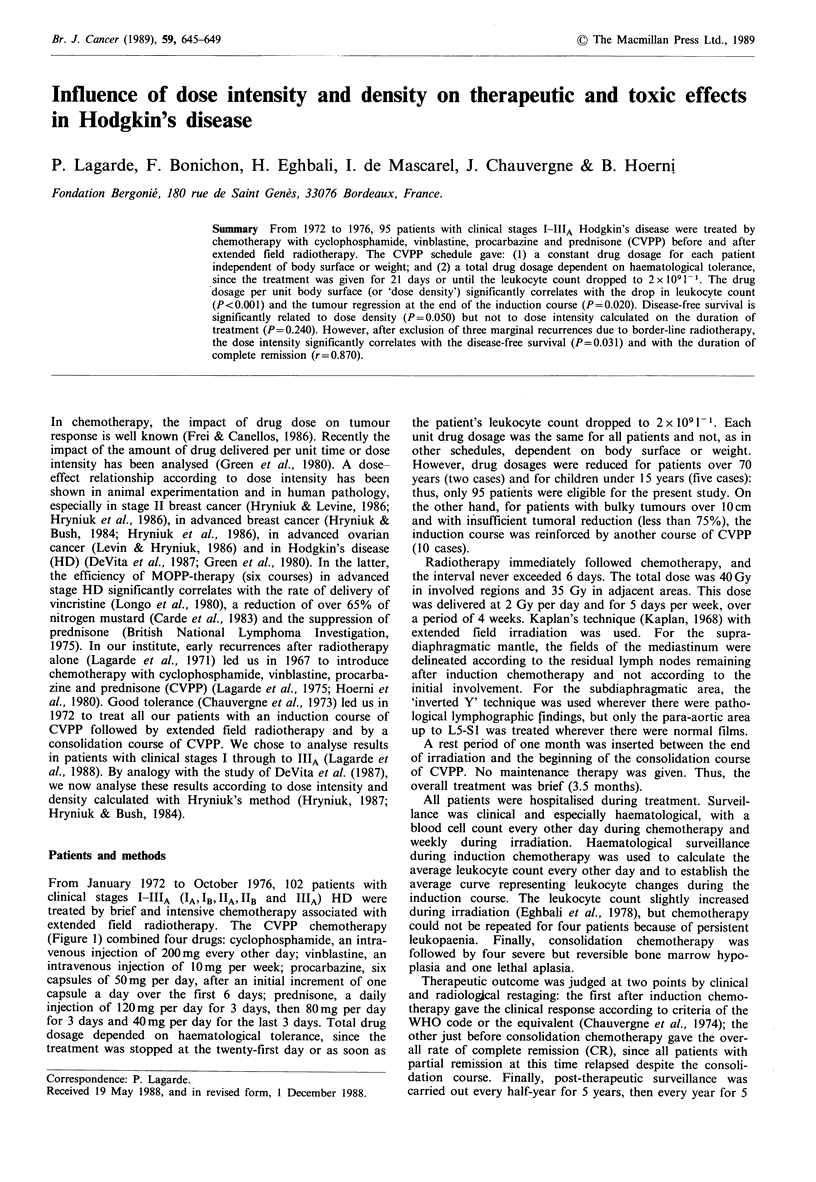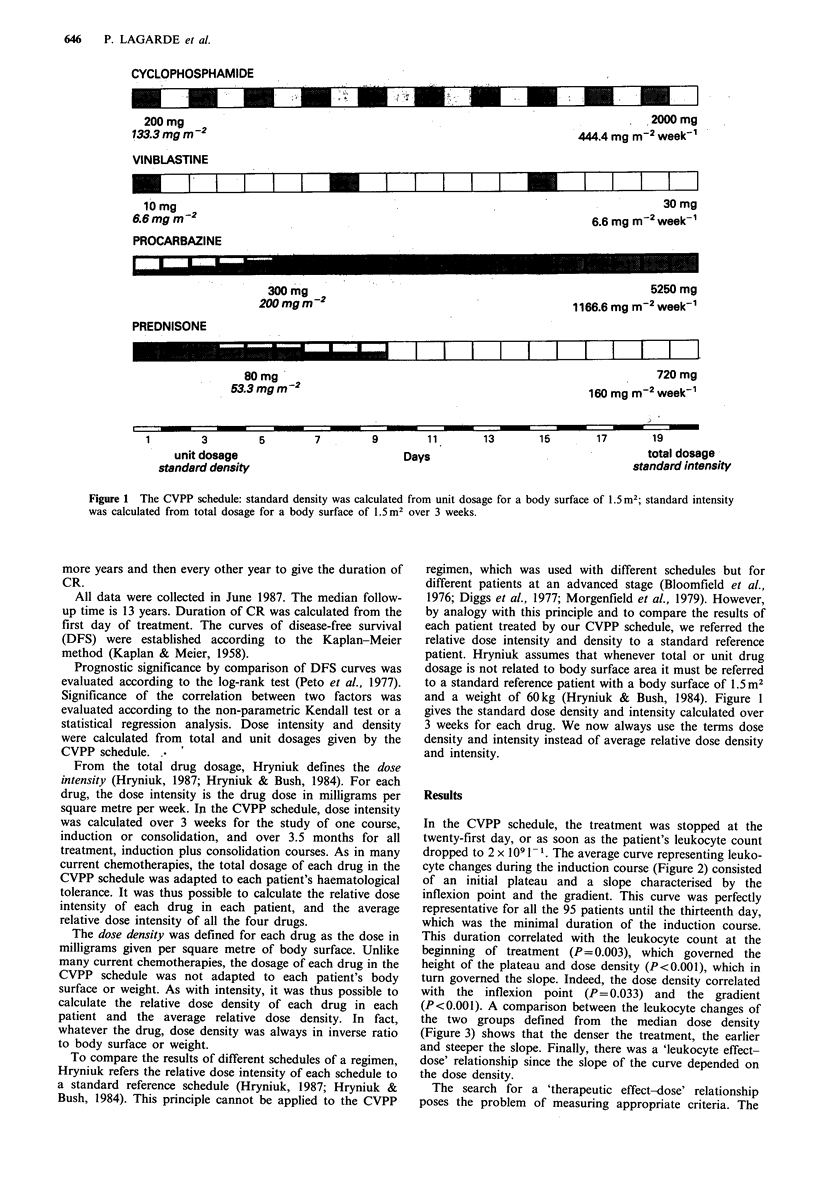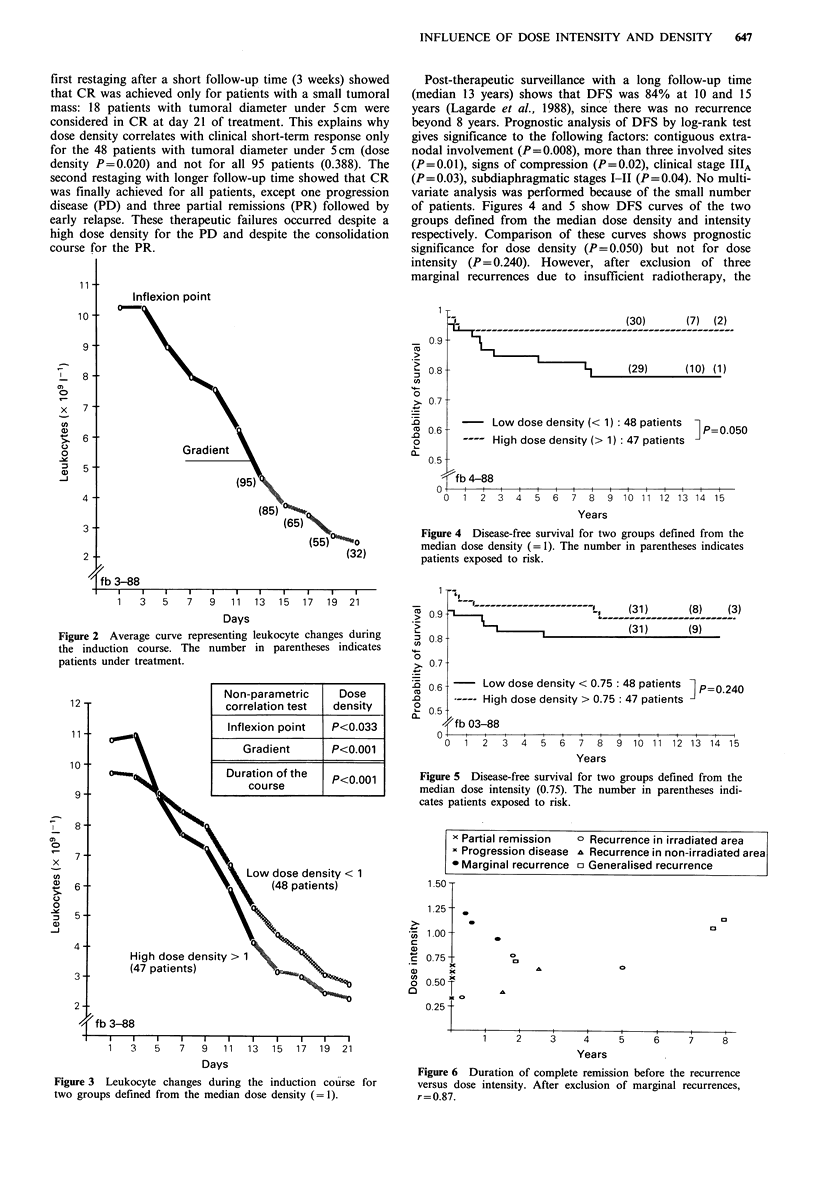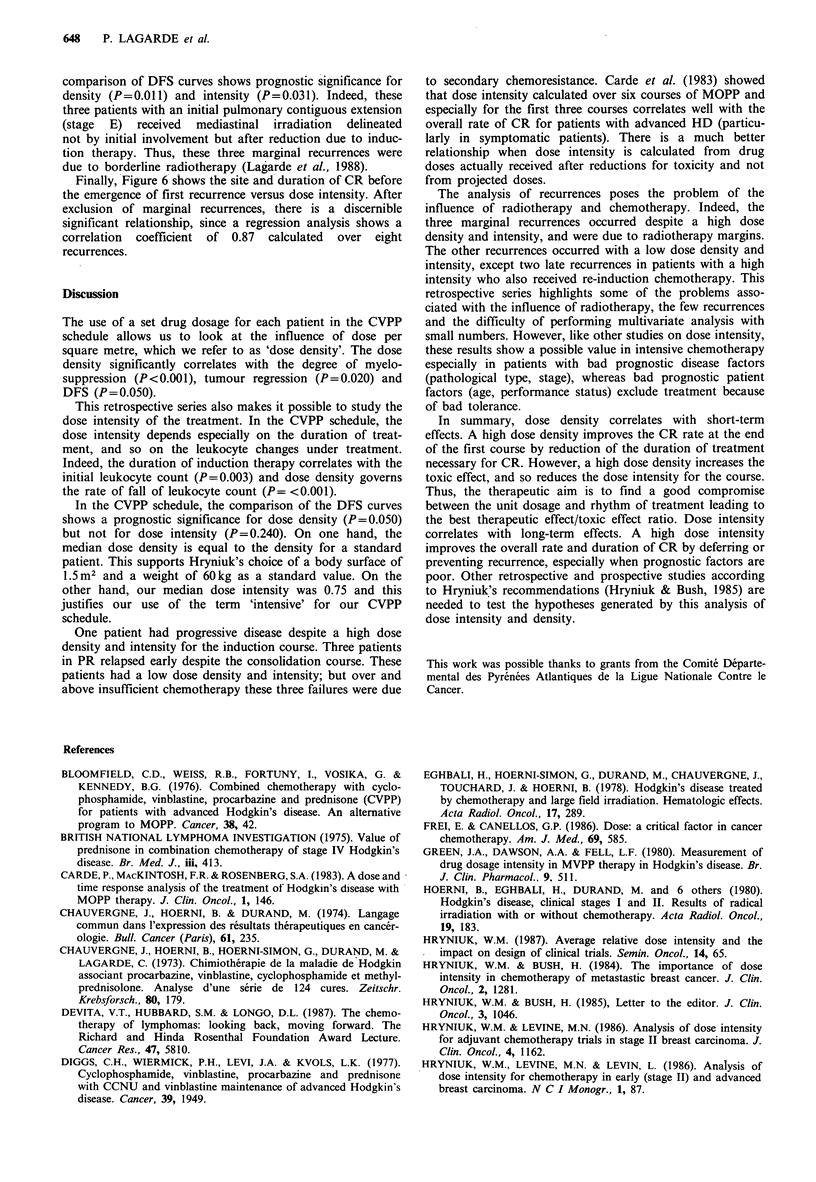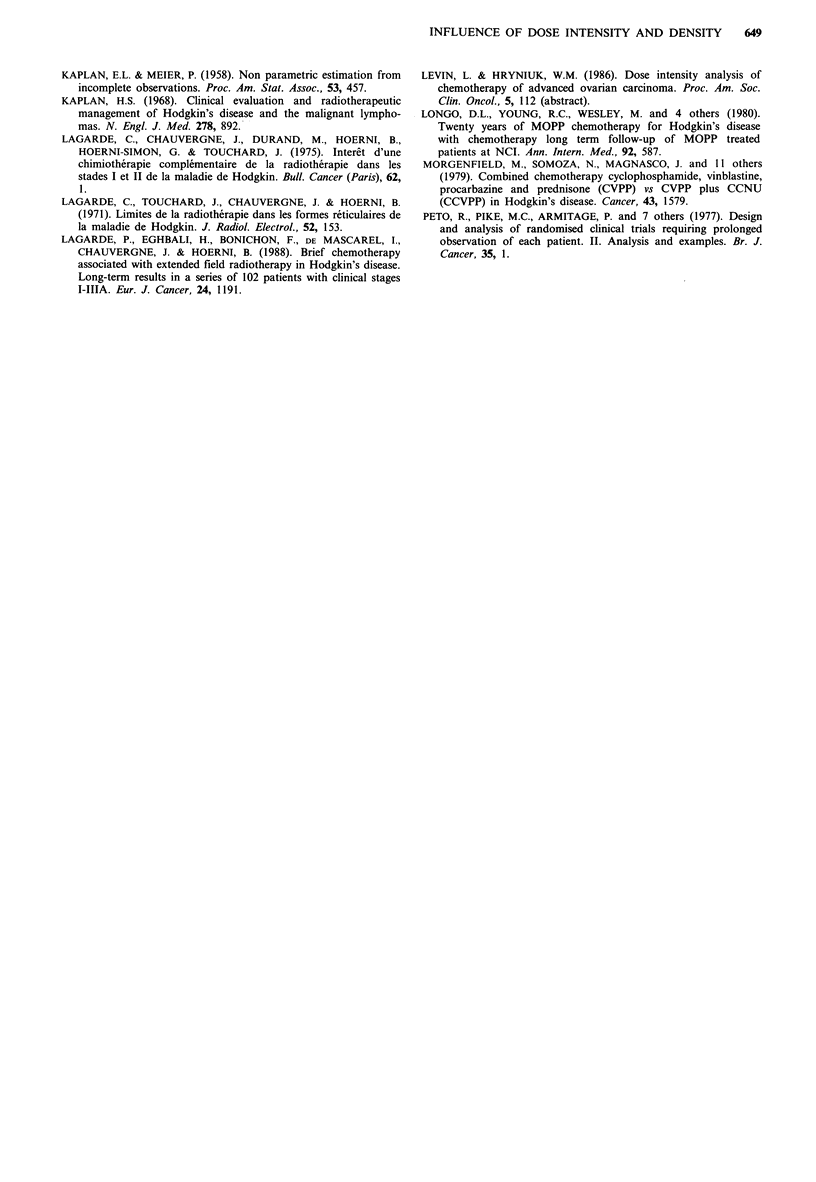# Influence of dose intensity and density on therapeutic and toxic effects in Hodgkin's disease.

**DOI:** 10.1038/bjc.1989.131

**Published:** 1989-04

**Authors:** P. Lagarde, F. Bonichon, H. Eghbali, I. de Mascarel, J. Chauvergne, B. Hoerni

**Affiliations:** Fondation BergoniÃ©, Bordeaux, France.

## Abstract

From 1972 to 1976, 95 patients with clinical stages I-IIIA Hodgkin's disease were treated by chemotherapy with cyclophosphamide, vinblastine, procarbazine and prednisone (CVPP) before and after extended field radiotherapy. The CVPP schedule gave: (1) a constant drug dosage for each patient independent of body surface or weight; and (2) a total drug dosage dependent on haematological tolerance, since the treatment was given for 21 days or until the leukocyte count dropped to 2 x 10(9) l-1. The drug dosage per unit body surface (or 'dose density') significantly correlates with the drop in leukocyte count (P less than 0.001) and the tumour regression at the end of the induction course (P = 0.020). Disease-free survival is significantly related to dose density (P = 0.050) but not to dose intensity calculated on the duration of treatment (P = 0.240). However, after exclusion of three marginal recurrences due to border-line radiotherapy, the dose intensity significantly correlates with the disease-free survival (P = 0.031) and with the duration of complete remission (r = 0.870).


*Br. J. Cancer* (1989), **59**, 645–649 © The Macmillan Press Ltd., 1989

### Influence of dose intensity and density on therapeutic and toxic effects in Hodgkin's disease

P. Lagarde, F. Bonichon, H. Eghbali, I. de Mascarel, J. Chauvergne & B. Hoerni

*Fondation Bergonié, 180 rue de Saint Genès, 33076 Bordeaux, France.*

**Summary** From 1972 to 1976, 95 patients with clinical stages I–$III_A$ Hodgkin's disease were treated by chemotherapy with cyclophosphamide, vinblastine, procarbazine and prednisone (CVPP) before and after extended field radiotherapy. The CVPP schedule gave: (1) a constant drug dosage for each patient independent of body surface or weight; and (2) a total drug dosage dependent on haematological tolerance, since the treatment was given for 21 days or until the leukocyte count dropped to $2 \times 10^9 l^{-1}$. The drug dosage per unit body surface (or 'dose density') significantly correlates with the drop in leukocyte count ($P<0.001$) and the tumour regression at the end of the induction course ($P=0.020$). Disease-free survival is significantly related to dose density ($P=0.050$) but not to dose intensity calculated on the duration of treatment ($P=0.240$). However, after exclusion of three marginal recurrences due to border-line radiotherapy, the dose intensity significantly correlates with the disease-free survival ($P=0.031$) and with the duration of complete remission ($r=0.870$).

In chemotherapy, the impact of drug dose on tumour response is well known (Frei & Canellos, 1986). Recently the impact of the amount of drug delivered per unit time or dose intensity has been analysed (Green *et al.*, 1980). A dose–effect relationship according to dose intensity has been shown in animal experimentation and in human pathology, especially in stage II breast cancer (Hryniuk & Levine, 1986; Hryniuk *et al.*, 1986), in advanced breast cancer (Hryniuk & Bush, 1984; Hryniuk *et al.*, 1986), in advanced ovarian cancer (Levin & Hryniuk, 1986) and in Hodgkin's disease (HD) (DeVita *et al.*, 1987; Green *et al.*, 1980). In the latter, the efficiency of MOPP-therapy (six courses) in advanced stage HD significantly correlates with the rate of delivery of vincristine (Longo *et al.*, 1980), a reduction of over 65% of nitrogen mustard (Carde *et al.*, 1983) and the suppression of prednisone (British National Lymphoma Investigation, 1975). In our institute, early recurrences after radiotherapy alone (Lagarde *et al.*, 1971) led us in 1967 to introduce chemotherapy with cyclophosphamide, vinblastine, procarbazine and prednisone (CVPP) (Lagarde *et al.*, 1975; Hoerni *et al.*, 1980). Good tolerance (Chauvergne *et al.*, 1973) led us in 1972 to treat all our patients with an induction course of CVPP followed by extended field radiotherapy and by a consolidation course of CVPP. We chose to analyse results in patients with clinical stages I through to $III_A$ (Lagarde *et al.*, 1988). By analogy with the study of DeVita *et al.* (1987), we now analyse these results according to dose intensity and density calculated with Hryniuk's method (Hryniuk, 1987; Hryniuk & Bush, 1984).

### Patients and methods

From January 1972 to October 1976, 102 patients with clinical stages I–$III_A$ ($I_A, I_B, II_A, II_B$ and $III_A$) HD were treated by brief and intensive chemotherapy associated with extended field radiotherapy. The CVPP chemotherapy (Figure 1) combined four drugs: cyclophosphamide, an intravenous injection of 200 mg every other day; vinblastine, an intravenous injection of 10 mg per week; procarbazine, six capsules of 50 mg per day, after an initial increment of one capsule a day over the first 6 days; prednisone, a daily injection of 120 mg per day for 3 days, then 80 mg per day for 3 days and 40 mg per day for the last 3 days. Total drug dosage depended on haematological tolerance, since the treatment was stopped at the twenty-first day or as soon as the patient's leukocyte count dropped to $2 \times 10^9 l^{-1}$. Each unit drug dosage was the same for all patients and not, as in other schedules, dependent on body surface or weight. However, drug dosages were reduced for patients over 70 years (two cases) and for children under 15 years (five cases): thus, only 95 patients were eligible for the present study. On the other hand, for patients with bulky tumours over 10 cm and with insufficient tumoral reduction (less than 75%), the induction course was reinforced by another course of CVPP (10 cases).

Radiotherapy immediately followed chemotherapy, and the interval never exceeded 6 days. The total dose was 40 Gy in involved regions and 35 Gy in adjacent areas. This dose was delivered at 2 Gy per day and for 5 days per week, over a period of 4 weeks. Kaplan's technique (Kaplan, 1968) with extended field irradiation was used. For the supradiaphragmatic mantle, the fields of the mediastinum were delineated according to the residual lymph nodes remaining after induction chemotherapy and not according to the initial involvement. For the subdiaphragmatic area, the 'inverted Y' technique was used wherever there were pathological lymphographic findings, but only the para-aortic area up to L5-S1 was treated wherever there were normal films.

A rest period of one month was inserted between the end of irradiation and the beginning of the consolidation course of CVPP. No maintenance therapy was given. Thus, the overall treatment was brief (3.5 months).

All patients were hospitalised during treatment. Surveillance was clinical and especially haematological, with a blood cell count every other day during chemotherapy and weekly during irradiation. Haematological surveillance during induction chemotherapy was used to calculate the average leukocyte count every other day and to establish the average curve representing leukocyte changes during the induction course. The leukocyte count slightly increased during irradiation (Eghbali *et al.*, 1978), but chemotherapy could not be repeated for four patients because of persistent leukopaenia. Finally, consolidation chemotherapy was followed by four severe but reversible bone marrow hypoplasia and one lethal aplasia.

Therapeutic outcome was judged at two points by clinical and radiological restaging: the first after induction chemotherapy gave the clinical response according to criteria of the WHO code or the equivalent (Chauvergne *et al.*, 1974); the other just before consolidation chemotherapy gave the overall rate of complete remission (CR), since all patients with partial remission at this time relapsed despite the consolidation course. Finally, post-therapeutic surveillance was carried out every half-year for 5 years, then every year for 5

Correspondence: P. Lagarde.
Received 19 May 1988, and in revised form, 1 December 1988.

**Figure 1** The CVPP schedule: standard density was calculated from unit dosage for a body surface of $1.5\,m^2$; standard intensity was calculated from total dosage for a body surface of $1.5\,m^2$ over 3 weeks.

more years and then every other year to give the duration of CR.

All data were collected in June 1987. The median follow-up time is 13 years. Duration of CR was calculated from the first day of treatment. The curves of disease-free survival (DFS) were established according to the Kaplan–Meier method (Kaplan & Meier, 1958).

Prognostic significance by comparison of DFS curves was evaluated according to the log-rank test (Peto *et al.*, 1977). Significance of the correlation between two factors was evaluated according to the non-parametric Kendall test or a statistical regression analysis. Dose intensity and density were calculated from total and unit dosages given by the CVPP schedule.

From the total drug dosage, Hryniuk defines the *dose intensity* (Hryniuk, 1987; Hryniuk & Bush, 1984). For each drug, the dose intensity is the drug dose in milligrams per square metre per week. In the CVPP schedule, dose intensity was calculated over 3 weeks for the study of one course, induction or consolidation, and over 3.5 months for all treatment, induction plus consolidation courses. As in many current chemotherapies, the total dosage of each drug in the CVPP schedule was adapted to each patient's haematological tolerance. It was thus possible to calculate the relative dose intensity of each drug in each patient, and the average relative dose intensity of all the four drugs.

The *dose density* was defined for each drug as the dose in milligrams given per square metre of body surface. Unlike many current chemotherapies, the dosage of each drug in the CVPP schedule was not adapted to each patient's body surface or weight. As with intensity, it was thus possible to calculate the relative dose density of each drug in each patient and the average relative dose density. In fact, whatever the drug, dose density was always in inverse ratio to body surface or weight.

To compare the results of different schedules of a regimen, Hryniuk refers the relative dose intensity of each schedule to a standard reference schedule (Hryniuk, 1987; Hryniuk & Bush, 1984). This principle cannot be applied to the CVPP regimen, which was used with different schedules but for different patients at an advanced stage (Bloomfield *et al.*, 1976; Diggs *et al.*, 1977; Morgenfield *et al.*, 1979). However, by analogy with this principle and to compare the results of each patient treated by our CVPP schedule, we referred the relative dose intensity and density to a standard reference patient. Hryniuk assumes that whenever total or unit drug dosage is not related to body surface area it must be referred to a standard reference patient with a body surface of $1.5\,m^2$ and a weight of 60 kg (Hryniuk & Bush, 1984). Figure 1 gives the standard dose density and intensity calculated over 3 weeks for each drug. We now always use the terms dose density and intensity instead of average relative dose density and intensity.

### Results

In the CVPP schedule, the treatment was stopped at the twenty-first day, or as soon as the patient's leukocyte count dropped to $2 \times 10^9\,l^{-1}$. The average curve representing leukocyte changes during the induction course (Figure 2) consisted of an initial plateau and a slope characterised by the inflexion point and the gradient. This curve was perfectly representative for all the 95 patients until the thirteenth day, which was the minimal duration of the induction course. This duration correlated with the leukocyte count at the beginning of treatment ($P=0.003$), which governed the height of the plateau and dose density ($P<0.001$), which in turn governed the slope. Indeed, the dose density correlated with the inflexion point ($P=0.033$) and the gradient ($P<0.001$). A comparison between the leukocyte changes of the two groups defined from the median dose density (Figure 3) shows that the denser the treatment, the earlier and steeper the slope. Finally, there was a 'leukocyte effect–dose' relationship since the slope of the curve depended on the dose density.

The search for a 'therapeutic effect–dose' relationship poses the problem of measuring appropriate criteria. The first restaging after a short follow-up time (3 weeks) showed that CR was achieved only for patients with a small tumoral mass: 18 patients with tumoral diameter under 5 cm were considered in CR at day 21 of treatment. This explains why dose density correlates with clinical short-term response only for the 48 patients with tumoral diameter under 5 cm (dose density $P=0.020$) and not for all 95 patients (0.388). The second restaging with longer follow-up time showed that CR was finally achieved for all patients, except one progression disease (PD) and three partial remissions (PR) followed by early relapse. These therapeutic failures occurred despite a high dose density for the PD and despite the consolidation course for the PR.

**Figure 2** Average curve representing leukocyte changes during the induction course. The number in parentheses indicates patients under treatment.

| Non-parametric correlation test | Dose density |
|---|---|
| Inflexion point | $P<0.033$ |
| Gradient | $P<0.001$ |
| Duration of the course | $P<0.001$ |

**Figure 3** Leukocyte changes during the induction course for two groups defined from the median dose density (=1).

Post-therapeutic surveillance with a long follow-up time (median 13 years) shows that DFS was 84% at 10 and 15 years (Lagarde *et al.*, 1988), since there was no recurrence beyond 8 years. Prognostic analysis of DFS by log-rank test gives significance to the following factors: contiguous extranodal involvement ($P=0.008$), more than three involved sites ($P=0.01$), signs of compression ($P=0.02$), clinical stage $III_A$ ($P=0.03$), subdiaphragmatic stages I–II ($P=0.04$). No multivariate analysis was performed because of the small number of patients. Figures 4 and 5 show DFS curves of the two groups defined from the median dose density and intensity respectively. Comparison of these curves shows prognostic significance for dose density ($P=0.050$) but not for dose intensity ($P=0.240$). However, after exclusion of three marginal recurrences due to insufficient radiotherapy, the

**Figure 4** Disease-free survival for two groups defined from the median dose density (=1). The number in parentheses indicates patients exposed to risk.

**Figure 5** Disease-free survival for two groups defined from the median dose intensity (0.75). The number in parentheses indicates patients exposed to risk.

**Figure 6** Duration of complete remission before the recurrence versus dose intensity. After exclusion of marginal recurrences, $r=0.87$.

comparison of DFS curves shows prognostic significance for density ($P=0.011$) and intensity ($P=0.031$). Indeed, these three patients with an initial pulmonary contiguous extension (stage E) received mediastinal irradiation delineated not by initial involvement but after reduction due to induction therapy. Thus, these three marginal recurrences were due to borderline radiotherapy (Lagarde et al., 1988).

Finally, Figure 6 shows the site and duration of CR before the emergence of first recurrence versus dose intensity. After exclusion of marginal recurrences, there is a discernible significant relationship, since a regression analysis shows a correlation coefficient of 0.87 calculated over eight recurrences.

### Discussion

The use of a set drug dosage for each patient in the CVPP schedule allows us to look at the influence of dose per square metre, which we refer to as 'dose density'. The dose density significantly correlates with the degree of myelosuppression ($P<0.001$), tumour regression ($P=0.020$) and DFS ($P=0.050$).

This retrospective series also makes it possible to study the dose intensity of the treatment. In the CVPP schedule, the dose intensity depends especially on the duration of treatment, and so on the leukocyte changes under treatment. Indeed, the duration of induction therapy correlates with the initial leukocyte count ($P=0.003$) and dose density governs the rate of fall of leukocyte count ($P=<0.001$).

In the CVPP schedule, the comparison of the DFS curves shows a prognostic significance for dose density ($P=0.050$) but not for dose intensity ($P=0.240$). On one hand, the median dose density is equal to the density for a standard patient. This supports Hryniuk's choice of a body surface of $1.5\,m^2$ and a weight of 60 kg as a standard value. On the other hand, our median dose intensity was 0.75 and this justifies our use of the term 'intensive' for our CVPP schedule.

One patient had progressive disease despite a high dose density and intensity for the induction course. Three patients in PR relapsed early despite the consolidation course. These patients had a low dose density and intensity; but over and above insufficient chemotherapy these three failures were due to secondary chemoresistance. Carde et al. (1983) showed that dose intensity calculated over six courses of MOPP and especially for the first three courses correlates well with the overall rate of CR for patients with advanced HD (particularly in symptomatic patients). There is a much better relationship when dose intensity is calculated from drug doses actually received after reductions for toxicity and not from projected doses.

The analysis of recurrences poses the problem of the influence of radiotherapy and chemotherapy. Indeed, the three marginal recurrences occurred despite a high dose density and intensity, and were due to radiotherapy margins. The other recurrences occurred with a low dose density and intensity, except two late recurrences in patients with a high intensity who also received re-induction chemotherapy. This retrospective series highlights some of the problems associated with the influence of radiotherapy, the few recurrences and the difficulty of performing multivariate analysis with small numbers. However, like other studies on dose intensity, these results show a possible value in intensive chemotherapy especially in patients with bad prognostic disease factors (pathological type, stage), whereas bad prognostic patient factors (age, performance status) exclude treatment because of bad tolerance.

In summary, dose density correlates with short-term effects. A high dose density improves the CR rate at the end of the first course by reduction of the duration of treatment necessary for CR. However, a high dose density increases the toxic effect, and so reduces the dose intensity for the course. Thus, the therapeutic aim is to find a good compromise between the unit dosage and rhythm of treatment leading to the best therapeutic effect/toxic effect ratio. Dose intensity correlates with long-term effects. A high dose intensity improves the overall rate and duration of CR by deferring or preventing recurrence, especially when prognostic factors are poor. Other retrospective and prospective studies according to Hryniuk's recommendations (Hryniuk & Bush, 1985) are needed to test the hypotheses generated by this analysis of dose intensity and density.

This work was possible thanks to grants from the Comité Départemental des Pyrénées Atlantiques de la Ligue Nationale Contre le Cancer.

### References

BLOOMFIELD, C.D., WEISS, R.B., FORTUNY, I., VOSIKA, G. & KENNEDY, B.G. (1976). Combined chemotherapy with cyclophosphamide, vinblastine, procarbazine and prednisone (CVPP) for patients with advanced Hodgkin's disease. An alternative program to MOPP. *Cancer*, **38**, 42.

BRITISH NATIONAL LYMPHOMA INVESTIGATION (1975). Value of prednisone in combination chemotherapy of stage IV Hodgkin's disease. *Br. Med. J.*, **iii**, 413.

CARDE, P., MACKINTOSH, F.R. & ROSENBERG, S.A. (1983). A dose and time response analysis of the treatment of Hodgkin's disease with MOPP therapy. *J. Clin. Oncol.*, **1**, 146.

CHAUVERGNE, J., HOERNI, B. & DURAND, M. (1974). Langage commun dans l'expression des résultats thérapeutiques en cancérologie. *Bull. Cancer (Paris)*, **61**, 235.

CHAUVERGNE, J., HOERNI, B., HOERNI-SIMON, G., DURAND, M. & LAGARDE, C. (1973). Chimiothérapie de la maladie de Hodgkin associant procarbazine, vinblastine, cyclophosphamide et methylprednisolone. Analyse d'une série de 124 cures. *Zeitschr. Krebsforsch.*, **80**, 179.

DEVITA, V.T., HUBBARD, S.M. & LONGO, D.L. (1987). The chemotherapy of lymphomas: looking back, moving forward. The Richard and Hinda Rosenthal Foundation Award Lecture. *Cancer Res.*, **47**, 5810.

DIGGS, C.H., WIERMICK, P.H., LEVI, J.A. & KVOLS, L.K. (1977). Cyclophosphamide, vinblastine, procarbazine and prednisone with CCNU and vinblastine maintenance of advanced Hodgkin's disease. *Cancer*, **39**, 1949.

EGHBALI, H., HOERNI-SIMON, G., DURAND, M., CHAUVERGNE, J., TOUCHARD, J. & HOERNI, B. (1978). Hodgkin's disease treated by chemotherapy and large field irradiation. Hematologic effects. *Acta Radiol. Oncol.*, **17**, 289.

FREI, E. & CANELLOS, G.P. (1986). Dose: a critical factor in cancer chemotherapy. *Am. J. Med.*, **69**, 585.

GREEN, J.A., DAWSON, A.A. & FELL, L.F. (1980). Measurement of drug dosage intensity in MVPP therapy in Hodgkin's disease. *Br. J. Clin. Pharmacol.*, **9**, 511.

HOERNI, B., EGHBALI, H., DURAND, M. and 6 others (1980). Hodgkin's disease, clinical stages I and II. Results of radical irradiation with or without chemotherapy. *Acta Radiol. Oncol.*, **19**, 183.

HRYNIUK, W.M. (1987). Average relative dose intensity and the impact on design of clinical trials. *Semin. Oncol.*, **14**, 65.

HRYNIUK, W.M. & BUSH, H. (1984). The importance of dose intensity in chemotherapy of metastastic breast cancer. *J. Clin. Oncol.*, **2**, 1281.

HRYNIUK, W.M. & BUSH, H. (1985), Letter to the editor. *J. Clin. Oncol.*, **3**, 1046.

HRYNIUK, W.M. & LEVINE, M.N. (1986). Analysis of dose intensity for adjuvant chemotherapy trials in stage II breast carcinoma. *J. Clin. Oncol.*, **4**, 1162.

HRYNIUK, W.M., LEVINE, M.N. & LEVIN, L. (1986). Analysis of dose intensity for chemotherapy in early (stage II) and advanced breast carcinoma. *N C I Monogr.*, **1**, 87.

KAPLAN, E.L. & MEIER, P. (1958). Non parametric estimation from incomplete observations. *Proc. Am. Stat. Assoc.*, **53**, 457.

KAPLAN, H.S. (1968). Clinical evaluation and radiotherapeutic management of Hodgkin's disease and the malignant lymphomas. *N. Engl. J. Med.* **278**, 892.

LAGARDE, C., CHAUVERGNE, J., DURAND, M., HOERNI, B., HOERNI-SIMON, G. & TOUCHARD, J. (1975). Interêt d'une chimiothérapie complémentaire de la radiothérapie dans les stades I et II de la maladie de Hodgkin. *Bull. Cancer (Paris)*, **62**, 1.

LAGARDE, C., TOUCHARD, J., CHAUVERGNE, J. & HOERNI, B. (1971). Limites de la radiothérapie dans les formes réticulaires de la maladie de Hodgkin. *J. Radiol. Electrol.*, **52**, 153.

LAGARDE, P., EGHBALI, H., BONICHON, F., DE MASCAREL, I., CHAUVERGNE, J. & HOERNI, B. (1988). Brief chemotherapy associated with extended field radiotherapy in Hodgkin's disease. Long-term results in a series of 102 patients with clinical stages I-IIIA. *Eur. J. Cancer*, **24**, 1191.

LEVIN, L. & HRYNIUK, W.M. (1986). Dose intensity analysis of chemotherapy of advanced ovarian carcinoma. *Proc. Am. Soc. Clin. Oncol.*, **5**, 112 (abstract).

LONGO, D.L., YOUNG, R.C., WESLEY, M. and 4 others (1980). Twenty years of MOPP chemotherapy for Hodgkin's disease with chemotherapy long term follow-up of MOPP treated patients at NCI. *Ann. Intern. Med.*, **92**, 587.

MORGENFIELD, M., SOMOZA, N., MAGNASCO, J. and 11 others (1979). Combined chemotherapy cyclophosphamide, vinblastine, procarbazine and prednisone (CVPP) *vs* CVPP plus CCNU (CCVPP) in Hodgkin's disease. *Cancer*, **43**, 1579.

PETO, R., PIKE, M.C., ARMITAGE, P. and 7 others (1977). Design and analysis of randomised clinical trials requiring prolonged observation of each patient. II. Analysis and examples. *Br. J. Cancer*, **35**, 1.